# Supplementary material for: TRIM25 promotes the cell survival and growth of hepatocellular carcinoma through targeting Keap1-Nrf2 pathway
Source: Nat Commun. 2020 Jan 17;11:348. doi: 10.1038/s41467-019-14190-2 (PMC6969153; doi:10.1038/s41467-019-14190-2)
Supplement: Supplementary file 1 — Supplementary Information [file 41467_2019_14190_MOESM1_ESM.pdf]

TRIM25 promotes the cell survival and growth of hepatocellular carcinoma through targeting Keap1-Nrf2 pathway

Liu *et al*.

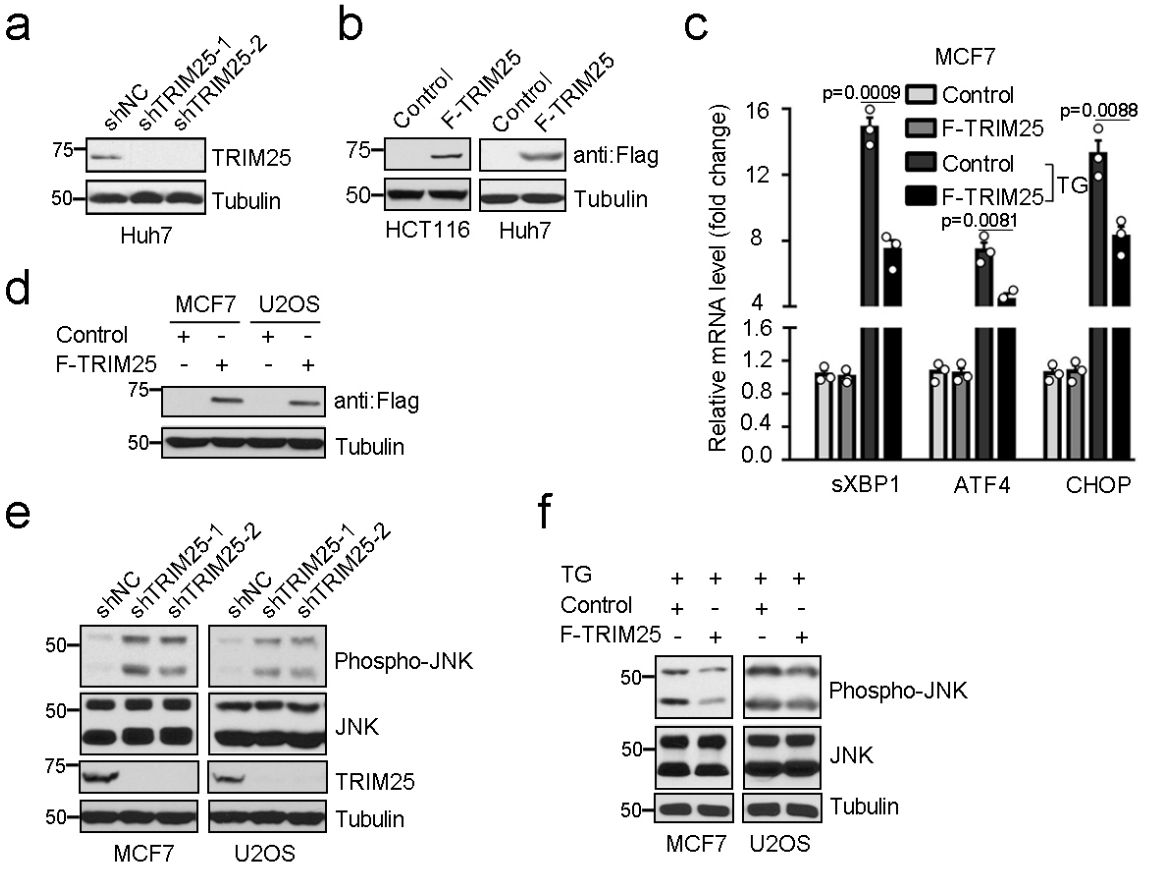

Supplementary Figure 1 | TRIM25 localizes at ER and suppresses UPR signaling
a Western blot analysis of the efficiency of the stable cell knocks down of TRIM25 in Huh7 cells. **b** Western blot analysis of HCT116 and Huh7 cells stably overexpressing control or F-TRIM25 vectors. **c** Relative mRNA fold change of UPR genes: sXBP1, ATF4, CHOP in MCF7 cells stably overexpressing control or F-TRIM25, treated with or without TG (1 μM) for 6 h. Data represent the mean ± SEM (n = 3). Statistical significance was assessed using two-tailed Student's t-test. **d** Western blot analysis of HCT116 and Huh7 cells stably overexpressing control or F-TRIM25 vectors in MCF7 and U2OS cells. **e** Western blot analysis of the levels of phospho-JNK/JNK in MCF7 and U2OS overexpressing control or F-TRIM25, treated with TG (1 μM) for 12 h. **f** Western blot analysis of the levels of phospho-JNK/JNK in MCF7 and U2OS cells after stable knock down of TRIM25.

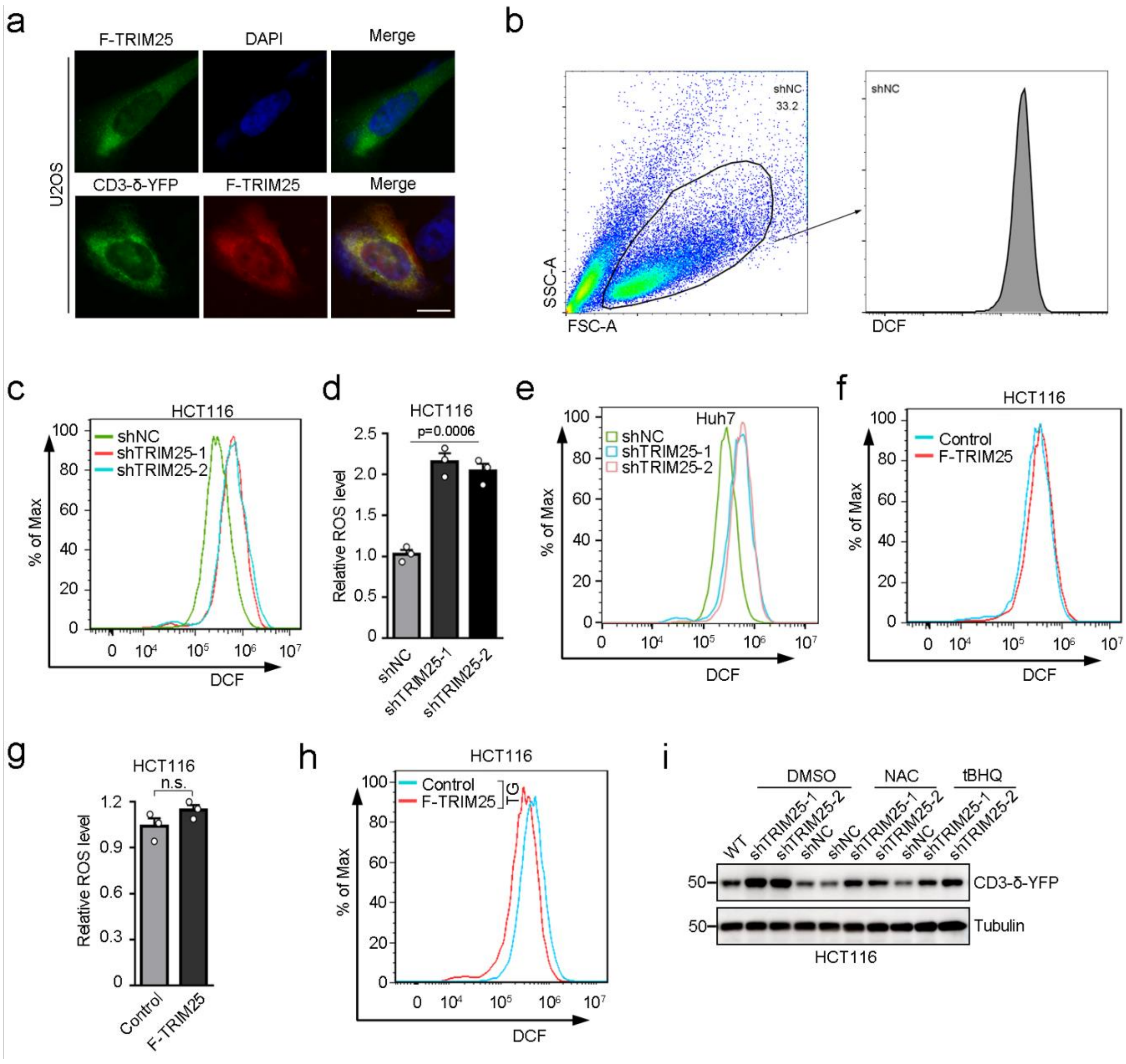

Supplementary Figure 2 | TRIM25 negatively regulates ROS

a Co-localization of F-TRIM25 (red) and ERAD substrate CD3-δ-YFP (green) in U2OS cells analyzed by fluorescence microscopy. TRIM25 was detected by anti-Flag antibody and the nucleus by DAPI (blue). Scale bar, 10 μm. **b** Gating strategy to determine the cellular ROS level presented on Fig 2f, g, j, k and Supplementary Fig 2c, e, f and h. **c, d** Flow cytometry analysis of ROS levels in HCT116 cells for control and stable knockdown of TRIM25 (**c**) quantified data is shown as (**d**). **e** Flow cytometry analysis of ROS levels in Huh7 cells stable knockdown of control or TRIM25. **f**, **g** Flow cytometry analysis of ROS levels in HCT116 cells stably overexpressing control or F-TRIM25 (**f**) quantified data is shown as (**g**). **h** Flow cytometry analysis of ROS levels in control and F-TRIM25-expressing HCT116 cells treated with TG (1 μM) for 6 h. **i** Western blot analysis of the levels of CD3-δ-YFP in HCT116 cells with control (shNC) or stable knockdown of TRIM25, treated with DMSO, NAC (2 mM) or tBHQ (20 μM) for 4h. For **d** and **g**, data represent the mean ± SEM (n = 3). Statistical significance was assessed using two-tailed Student's t-test. n.s. not significant.

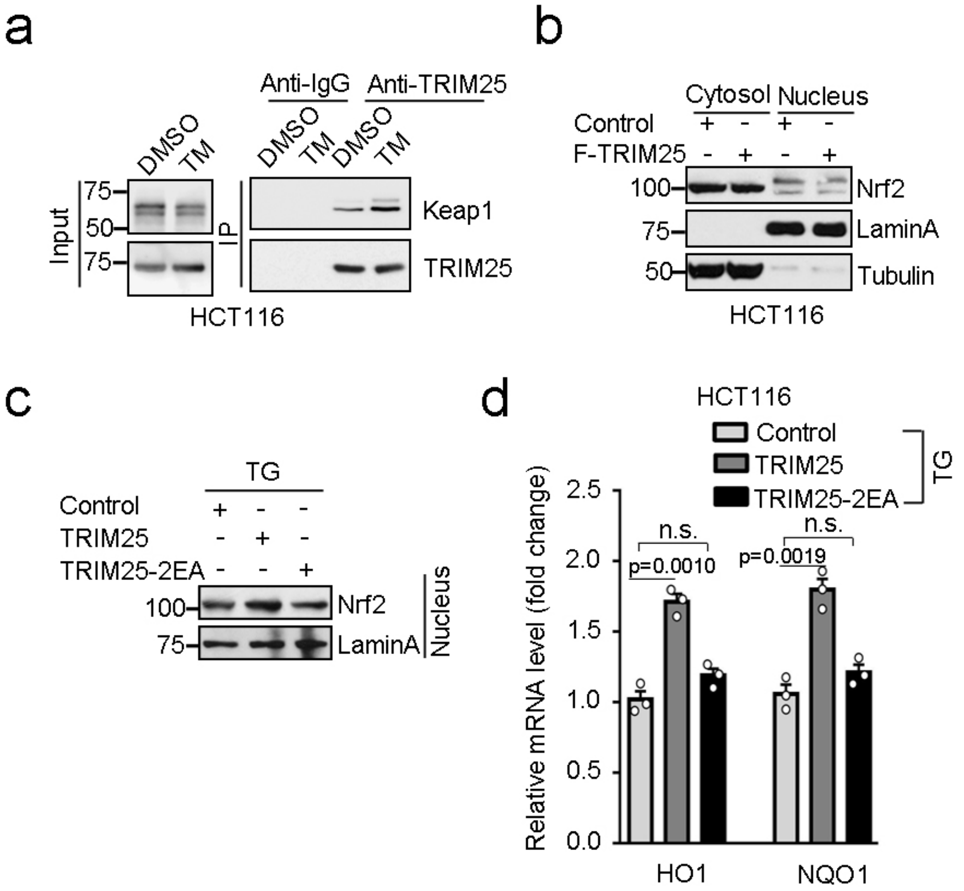

Supplementary Figure 3 | The E3 ubiquitin activity of TRIM25 is required for Nrf2 regulation

a Co-immunoprecipitation (co-IP) assay analyzes the interaction of endogenous TRIM25 and Keap1 in HCT116 cells, treated with or without TM (5 µg/ml) for 12 h. Cell lysates containing comparable amounts of TRIM25 or Keap1 were analyzed for the TRIM25-Keap1 interaction. **b** Nuclear/cytosolic fractionation assay and western blot analysis of the level of Nrf2 in control and F-TRIM25-expressing HCT116 cells. LaminA is an internal control for nuclear fraction. **c** The level of Nrf2 in the nuclear fraction of HCT116 cells with control, TRIM25-2EA or TRIM25-expressing, treated with TG (1 µM) for 12 h. **d** Relative mRNA fold change of Nrf2 downstream genes HO1, NQO1 in HCT116 cells expressing control, TRIM25 or TRIM25-2EA, treated with TG (1 µM) for 12 h. Data represent the mean ± SEM (n = 3). Statistical significance was assessed using two-tailed Student's t-test. n.s. not significant.

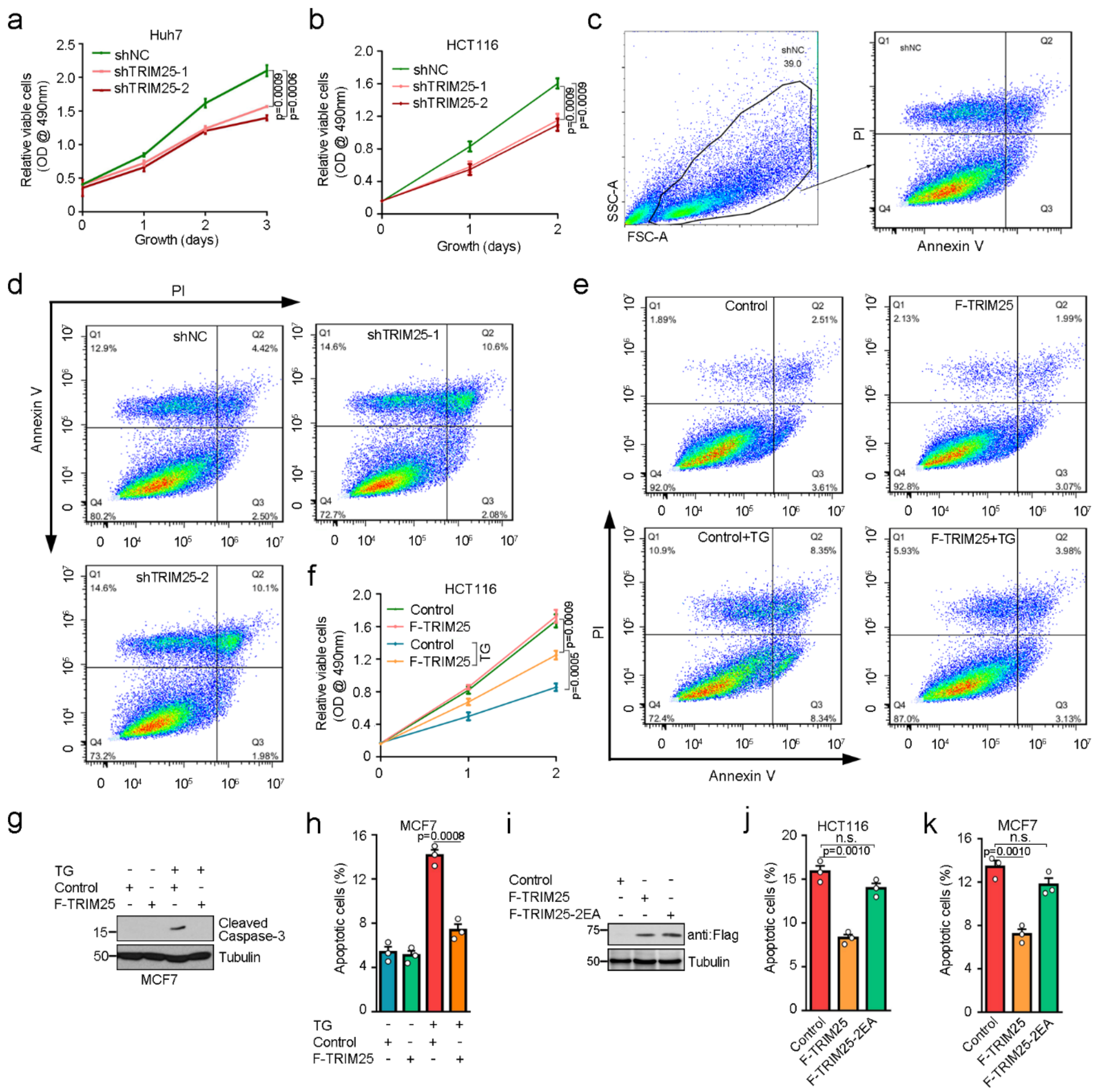

Supplementary Figure 4 | TRIM25 negatively regulates tumor cell apoptosis

a Cell growth of Huh7 cells in control (shNC) or after stable knockdown of TRIM25. Data represent the mean ± SEM (n=6). **b** Cell growth of HCT116 cells in control (shNC) or after stable knockdown of TRIM25. Data represent the mean ± SEM (n=6). **c** Gating strategy to determine the percentage of apoptotic cells presented on Fig 5c, d, g, h, k, m and Supplementary Fig 4d, e, h, j and k. **d** Representative flow cytometry analysis of apoptosis in HCT116 cells stable knockdown of control (shNC) or TRIM25. **e** Representative flow cytometry analysis of apoptotic HCT116 cells expressing control and F-TRIM25, treated with or without TG (1 μM) for 12 h. **f** Cell growth of control and F-TRIM25-expressing HCT116 cells treated with or without TG (1 μM). Data represent the mean ± SEM (n=6). **g** Western blot analysis of the levels of cleaved-caspase3 in control and F-TRIM25-expressing MCF7 cells treated with or without TG (1 μM) for 12 h. **h** Quantification of apoptotic MCF7 cells expressing control and F-TRIM25, treated with or without TG (1 μM) for 12 h. **i** Western blot analysis of HCT116 cells stably overexpressing control, F-TRIM25 or TRIM25-2EA. **j, k** Quantification of apoptotic HCT116 (**j**) and MCF7 (**k**) cells expressing control, TRIM25 or

TRIM25-2EA, treated with TG (1 μM) for 12 h. For **a, b, f, h, j** and **k**, data represent the mean ± SEM (n = 3, unless otherwise indicated). Statistical significance was assessed using two-tailed Student's t-test. n.s. not significant.

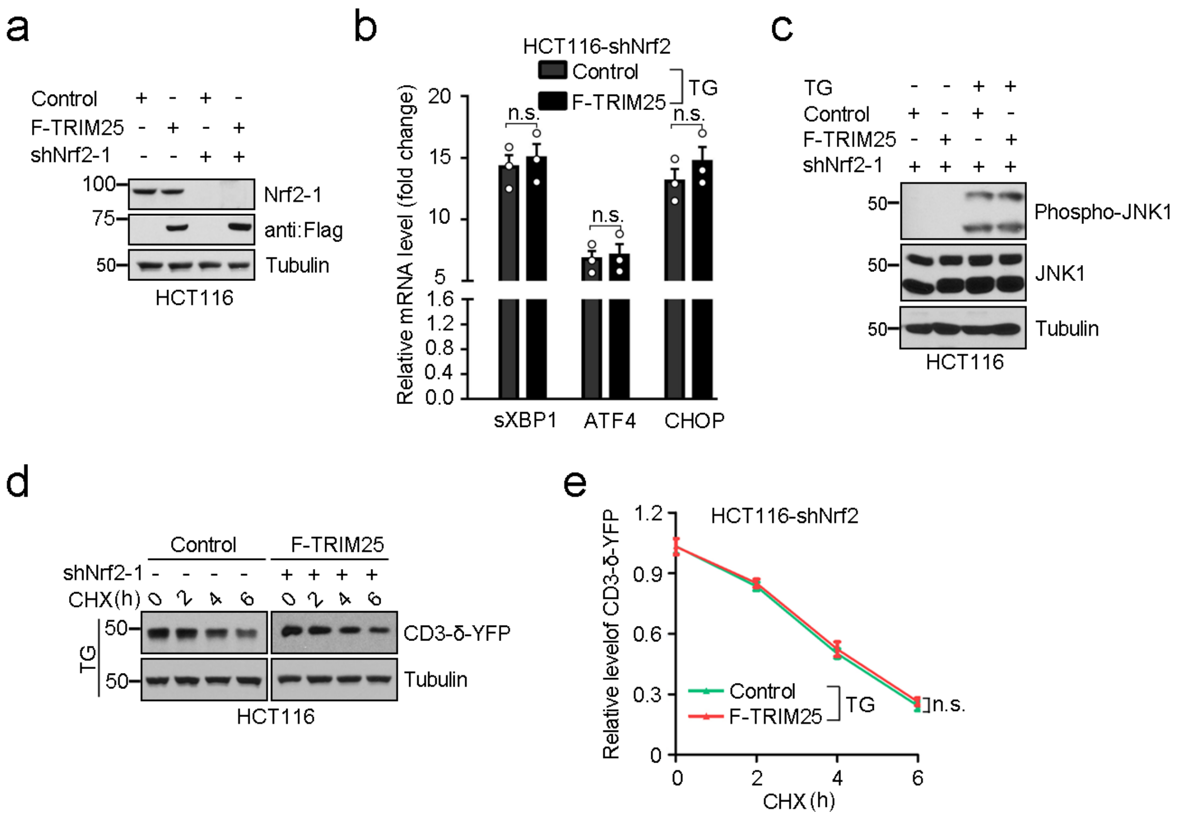

Supplementary Figure 5 | Nrf2 is required for TRIM25 mediated inhibition of UPR signaling pathways and ERAD

a Western blot analysis the levels of Nrf2 in HCT116 cells stably knocking down control or Nrf2, and in these cells simultaneously stably expressing control or F-TRIM25 and analysis of Nrf2 and F-TRIM25. **b** Relative mRNA fold change of UPR genes: sXBP1, ATF4, CHOP in control and F-TRIM25-expressing HCT116 cells with stable knock down of Nrf2. **c** Western blot analysis of the levels of phospho-JNK/JNK in control and F-TRIM25-expressing HCT116 cells with stable knock down of Nrf2, treated with or without TG (1 μM) for 12 h. **d, e** Half-life of CD3-δ-YFP in control and F-TRIM25-expressing HCT116 cells with stable knock down of Nrf2, pre-treated with TG (1 μM) for 6 h and then treated with cycloheximide (CHX) at the indicated times and analyzed by western blot. Representative western blot (**d**) and the corresponding quantified graph (**e**) are shown. For **b** and **e**, data represent the mean ± SEM (n = 3). Statistical significance was assessed using two-tailed Student’s t-test. n.s. not significant.

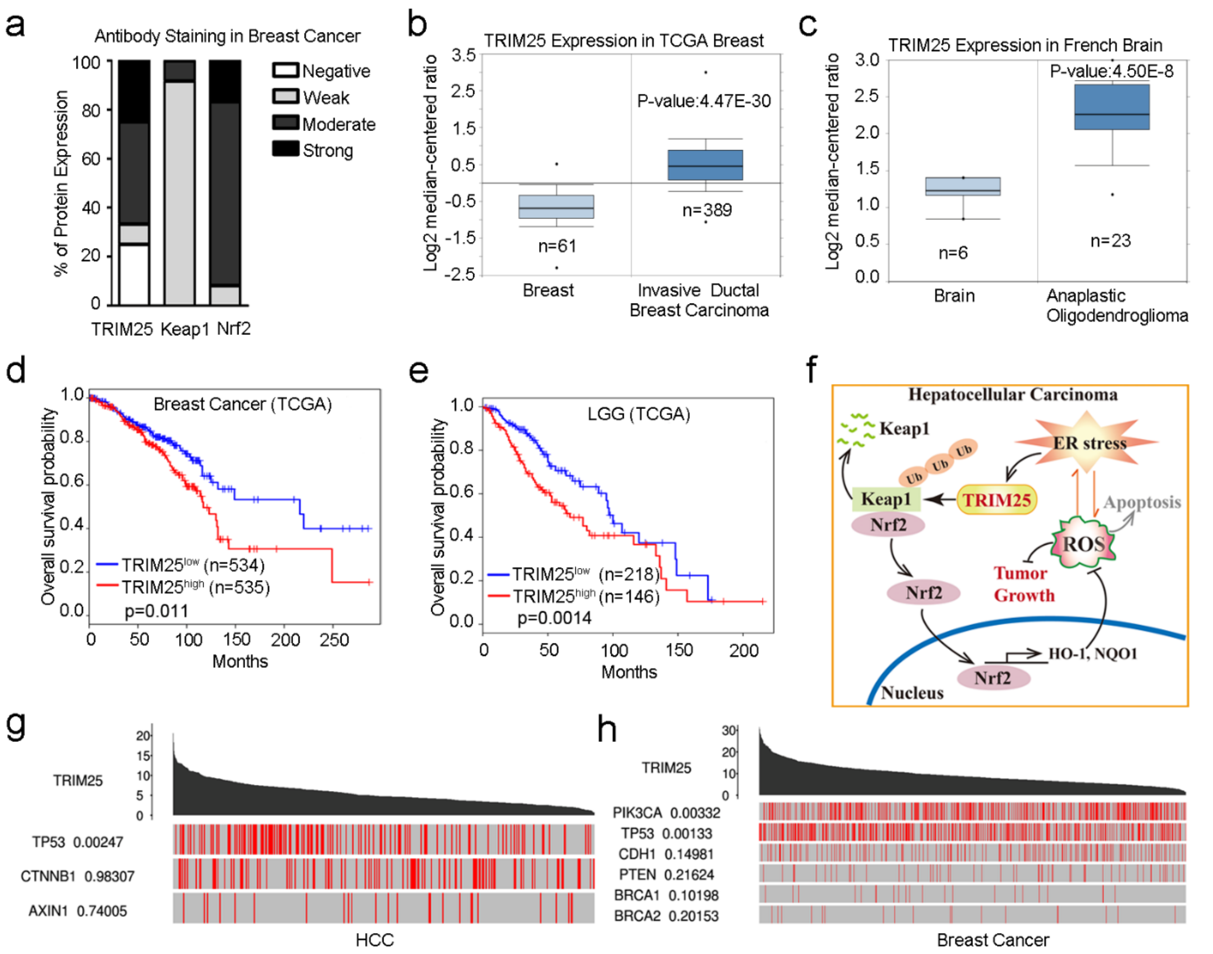

Supplementary Figure 6 | Analysis of expression of TRIM25 in different tumor tissues

a The percentage of TRIM25, Keap1 and Nrf2 mRNA expression levels (grouped as negative, weak, moderate and strong) were analyzed from online database (https://www.proteinatlas.org/) in human breast cancer tissues. **b**, **c** Comparison of the TRIM25 mRNA levels analyzed from the online database (https://www.oncomine.org/) in human breast cancers (**b**), French Brain cancers (**c**) and its corresponding normal tissues. The images are plotted on a log2 scale and centered on the median of its expression levels. For **b**, normal tissues/Invasive ductal breast carcinoma tissues: n=61/389, maximum=0.505/3.002, median=-0.681/0.449, minimum=-2.31/-1.071. For **c**, normal tissues/Anaplastic oligodendroglioma tissues: n=6/23, maximum=1.404/2.991, median=1.23/2.257, minimum=0.843/1.169. Statistical significance was assessed using two-tailed Student's t-test. **d, e** The overall survival and disease-free survival probability were compared between TRIM25 high and low expression in breast cancer and brain lower grade glioma (LGG) patients from TCGA database. The statistical significance was assessed using two-sided log-rank test, log-rank *p* values were shown. **f** A schematic model illustrating that TRIM25 regulates Keap1/Nrf2/ROS signaling pathway upon ER stress to promote HCC survival. TRIM25 is up-regulated during ER stress, which facilitates HCC cell survival by targeting Keap1 for ubiquitination and degradation, leading to Nrf2 translocated into the nucleus, activated Nrf2 downstream genes and reduced ROS levels. **g, h** The relationship of TRIM25 expression between driver mutated (red) and not-mutated (gray) samples in the HCC (**g**) and Breast cancer (**h**) were analyzed from the online database (http://tumorsurvival.org/).

Statistical significance was assessed using permutation test.

Supplementary Figure 7 | Uncropped western blot images related to Figure 1

Figure 1b

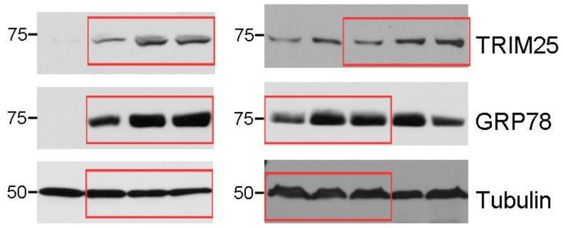

Figure 1f

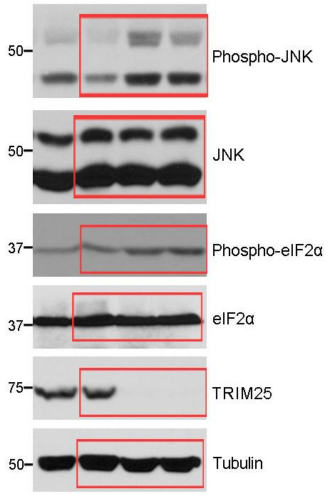

Figure 1g

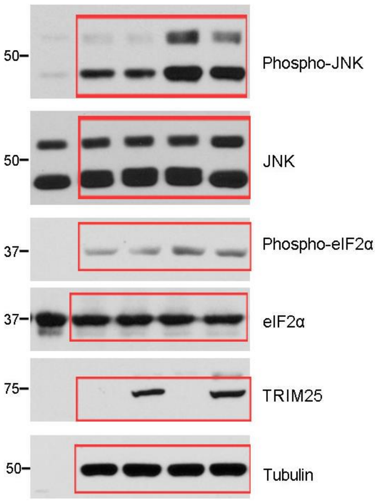

Figure 1h

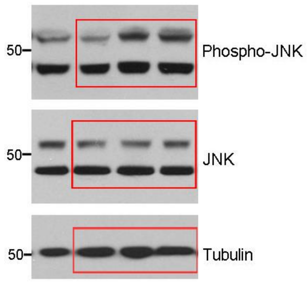

Figure 1i

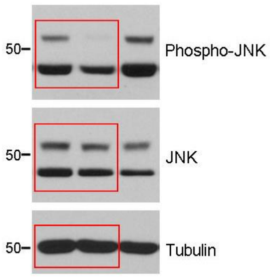

Supplementary Figure 8 | Uncropped western blot images related to Figure 2

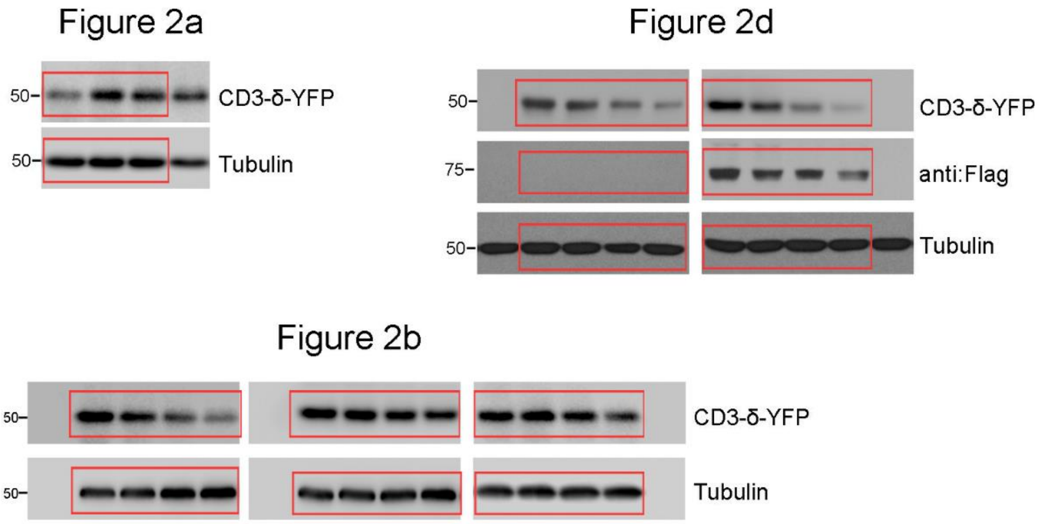

Supplementary Figure 9 | Uncropped western blot images related to Figure 3

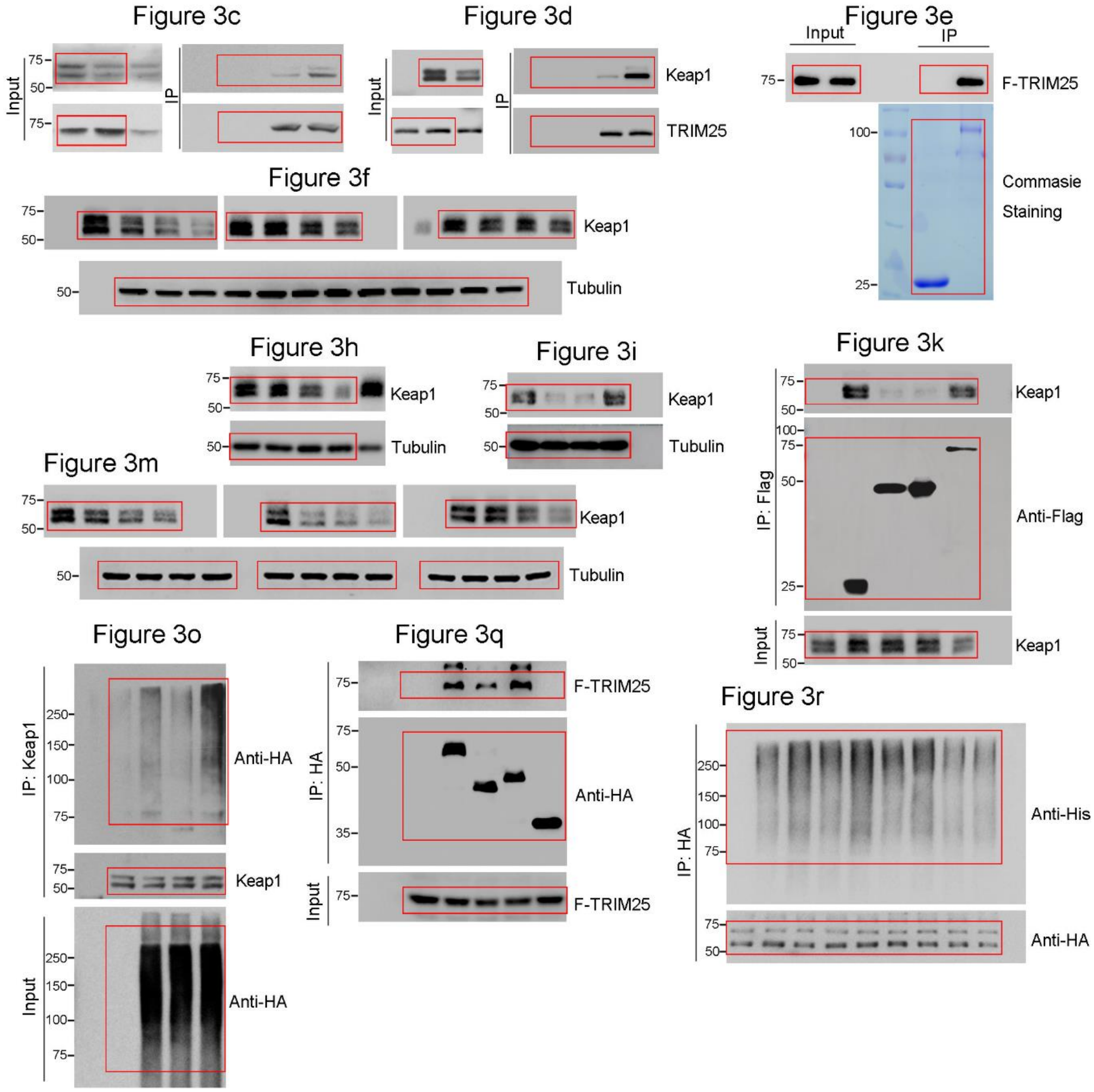

Supplementary Figure 10 | Uncropped western blot images related to Figure 4

Figure 4a

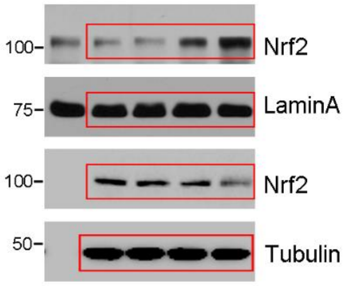

Figure 4b

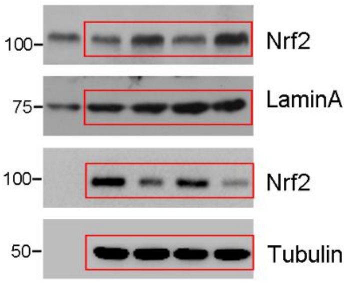

Figure 4c

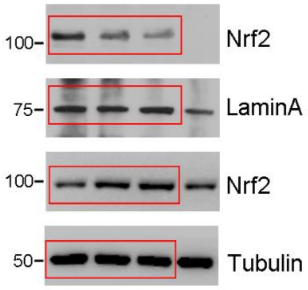

Figure 4d

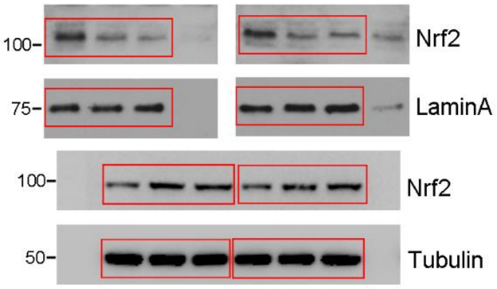

Supplementary Figure 11 | Uncropped western blot images related to Figure 5

Figure 5a

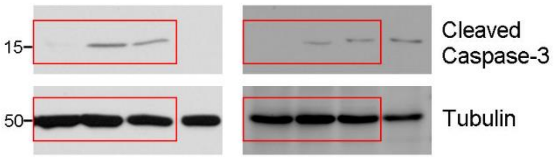

Figure 5b

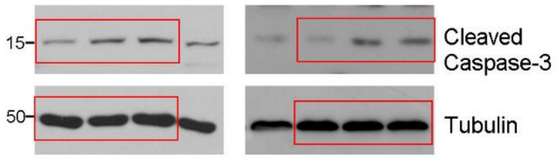

Figure 5e

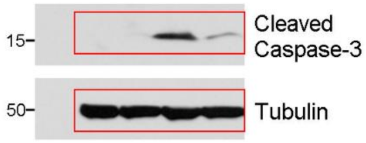

Figure 5f

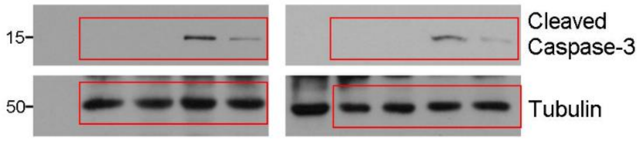

Figure 5i

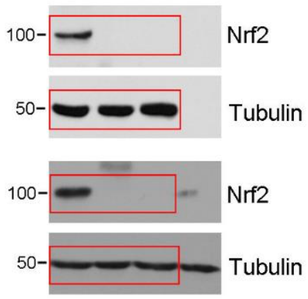

Figure 5l

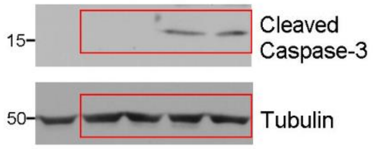

Supplementary Table 1. Primer sequences for quantitative real-time PCR.

qRT-PCR primers for detecting ER stress and ROS related genes	
BiP-F	TGTTCAACCAATTATCAGCAAACTC
BiP-R	TTCTGCTGTATCCTCTTCACCAGT
sXBP1-F	CTGAGTCCGAATCAGGTGCAG
sXBP1-R	ATCCATGGGGAGATGTTCTGG
ATF4-F	GTTCTCCAGCGACAAGGCTA
ATF4-R	ATCCTGCTTGCTGTTGTTGG
CHOP-F	AGAACCAGGAAACGGAAACAGA
CHOP-R	TCTCCTTCATGCGCTGCTTT
HO1-F	ACATCGACAGCCCCACCAAGTTCAA
HO1-R	CTGACGAAGTGACGCCATCTGTGAG
NQO1-F	GGATTGGACCGAGCTGGAA
NQO1-R	AATTGCAGTGAAGATGAAGGCAAC
qRT-PCR primers for detecting all of TRIMs	
TRIM1-F	CCAGCCTCCGTGGTTCTTAAT
TRIM1-R	ACAGGTCAATTCAGACTCCAGT
TRIM2-F	TGCGCCAGATTGACAAGCA
TRIM2-R	GCACCTCTCGCAGAAAGTG
TRIM3-F	GCGACCTGGAGACCATTTGT
TRIM3-R	GCTACTGCCGATGTGTTCCTG
TRIM4-F	TGAGAATCAGCACGGAGTTTTC
TRIM4-R	CCTGGTCAACACTTCTTTTGGA
TRIM5-F	AAGTCCATGCTAGACAAAGGAGA
TRIM5-R	GTTGGCTACATGCCGATTAGG
TRIM6-F	ACTGGGTTGACGTGACCCT
TRIM6-R	TCCCACAAACCTCACTTGTCT
TRIM7-F	TCCATGTTCAAGTCCCTCTCC
TRIM7-R	GGCCAGGTTCTCATTCTGCT
TRIM8-F	CGTGGAGATCCGAAGGAATGA
TRIM8-R	CAGGCGCTTGTCTGACTCG
TRIM9-F	GTGTGCGGCTCCTTCTATCG
TRIM9-R	GCTGTATAGGCTCATCTTGTCCA
TRIM10-F	CTGCCCCATCTGTCAGGGTA
TRIM10-R	GGTATCTCACAGTAGCGGGTAA
TRIM13-F	GTTTTGCCTTGCTCCCACAAC
TRIM13-R	TCCTTACGGCATGTAGGACAC
TRIM14-F	TGAAGGGGAAATTCACTGAACTC
TRIM14-R	AGCCTCTGGACAGGATCGG
TRIM15-F	TCCCTGAAGGTGGTCCATGAG

TRIM15-R	CAGGATCTTGCCCGAGGATT
TRIM16-F	GTCCTGTCTAACCTGCATGGT
TRIM16-R	GGCAGTATCGCCAGTTGTG
TRIM18-F	CTGACCTGCCCTATTTGTCTG
TRIM18-R	GCACAGTGTGATACTAGGATGC
TRIM20-F	TAAGACCCCTAGTGACCATCTG
TRIM20-R	TTCCCCATAGTAGGTGACCAG
TRIM22-F	CTGTCCTGTGTGTCAGACCAG
TRIM22-R	TGTGGGCTCATCTTGACCTCT
TRIM23-F	TGGTTGTAAACAAGCTCGGAG
TRIM23-R	ACTCTAGCACCTTCACTACAGC
TRIM25-F	AATCGGCTGCGGGAATTTTTC
TRIM25-R	TCTCACATCATCCAGTGCTCT
TRIM26-F	TGCACTACTACTGTGAGGACG
TRIM26-R	TCCTTAGGGTACTCAGGTGGT
TRIM28-F	TTTCATGCGTGATAGTGGCAG
TRIM28-R	GCCTCTACACAGGTCTCACAC
TRIM29-F	CTGTTCGCGGGCAATGAGT
TRIM29-R	TGCCTTCCATAGAGTCCATGC
TRIM31-F	AACCTGTCACCATCGACTGTG
TRIM31-R	TGATTGCGTTCTTCCTTACGG
TRIM33-F	ATGTGGAGAGTGGCTATGTAAGA
TRIM33-R	GGGCGTTGACCAGATGCTC
TRIM34-F	GACGCTGGATAAGTTTGCAGA
TRIM34-R	CCACCCATACGCACCATTTC
TRIM35-F	TGAAGGAGGACGACGTTTCTT
TRIM35-R	GCCCAGGTACTTGCAGACATC
TRIM36-F	GAGCTGTTTACCCACCCATTG
TRIM36-R	CTGATCCCACATCGTTGAATGA
TRIM38-F	GAGCCTGATGACGAACCCAG
TRIM38-R	TCTTGATCCGTCTCTTTGAGGG
TRIM39-F	GAAAGGGCGAGTTGACTCCAC
TRIM39-R	GGCTGCATATTTGTCCCCATT
TRIM40-F	ACATCTCTTCTGTCGAGTGTGC
TRIM40-R	GGCAGATATAGCCTGTCCCTA
TRIM41-F	CTGCCGAGTTTGTGTAACCCA
TRIM41-R	CTCCTCCATGTCACCCTCGTA
TRIM42-F	GGCCAATGATCCCAACTGTAA
TRIM42-R	AGCAGCGGCTCTCATAGTAGT
TRIM43-F	AGGGAACCATCACCGAAAATG

TRIM43-R	TTGTTTGCCTATGGGTCCCAC
TRIM44-F	CCATCTGGCCGAATACGTCC
TRIM44-R	TGCCTCGCTTTCTATCTCCCT
TRIM45-F	AACTCAGGCAAGACTCACTGC
TRIM45-R	CCCTCGGATGTCCACTACTG
TRIM46-F	TTCCGACCCAAGGGCCTTAT
TRIM46-R	AGAGTTGACATACCAGGCGTT
TRIM47-F	CTGAGCAGTCCAAAGTCCTGA
TRIM47-R	CTACGGCTGCACTCTTGATG
TRIM48-F	GGATGTGACCGTCAAAATCCG
TRIM48-R	CACCCAAGACTAAAGAGTCCCT
TRIM49-F	ATCTTACAGGTCTTTCAGGGGG
TRIM49-R	GCACTGGACAAGAAATGGGAT
TRIM50-F	GGCCCTTAGAAGGCGCATT
TRIM50-R	GCAGGGTCCAACTTGAGAGG
TRIM51-F	GGCCCTGTTTGTACCTCAACT
TRIM51-R	TTCTCTGCCGCGTTGTCTTC
TRIM52-F	ATGGCTGGTTATGCCACTACT
TRIM52-R	CTCGTCCTCCTTACTCCACAG
TRIM54-F	ATCGTGCAGGCATGAGGTTG
TRIM54-R	CCTCGCACATGAGGTGCTG
TRIM55-F	TTGTCAGCACAACCTGTGTAG
TRIM55-R	CCCATGTCTATCCAAAACCACTT
TRIM56-F	GCCTGCATACCTACTGCCAAG
TRIM56-R	GCAGCCCATTGACGAAGAAGT
TRIM58-F	TACCAGGTAAAGCTCCAGATGG
TRIM58-R	GAAAGCCACGATGCTTCTCAA
TRIM60-F	CTCCAAGAGGAGTCTAGCTGT
TRIM60-R	TCCTTCCAGGATACACTGAGG
TRIM61-F	TCTGTCAGAAAGACCTAGAGCTT
TRIM61R	CACGGTGCATTGTATTCCTCC
TRIM62-F	CGAGCAGCATCAGGTCACC
TRIM62-R	CCAGTTGTCGCTTGAGCAG
TRIM63-F	CTTCCAGGCTGCAAATCCCTA
TRIM63-R	ACACTCCGTGACGATCCATGA
TRIM65-F	CGCCAACCGTCACTTCTATCT
TRIM65-R	ACAGGGTCAGGGTCCTACC
TRIM66-F	TCCCGGAGACTTCACCTTGTA
TRIM66-R	GTTCCACCACTAGGCAGCTAT
TRIM67-F	CGTGTCCCGAGCATGAAATG

TRIM67-R	CCTGAGATAGTTGTGCCTTGTG
TRIM68-F	GGAGCCCATGAGCATTGACT
TRIM68-R	GACAGGTGTAACCCCAGTTCT
TRIM69-F	CTTGCCATCCAACAGGGTCAA
TRIM69-R	TTCCTTGTGAGCAGCAATAGC
TRIM71-F	GTACCCATCTGTCGTGAGTGC
TRIM71-R	CGGCTTTGACCTCCGACTG
TRIM72-F	GAGGTGGATGTTGGCGACAA
TRIM72-R	CCGAAGCTCAGGTAAAGGC
TRIM73-F	CAGGAGCAGAAGAAGGTGGATG
TRIM73-R	CCGCACTGTAGCATTAGGGACT
TRIM74-F	CAGGAGCAGAAGAAGGTGGATG
TRIM74-R	AACTCATGGTGGTCCTCATTTCC
