# Supplementary material for: TRIM25 promotes the cell survival and growth of hepatocellular carcinoma through targeting Keap1-Nrf2 pathway
Source: Nat Commun. 2020 Jan 17;11:348. doi: 10.1038/s41467-019-14190-2 (PMC6969153; doi:10.1038/s41467-019-14190-2)
Supplement: Supplementary file 3 — Reporting Summary [file 41467_2019_14190_MOESM3_ESM.pdf]

nature research

Corresponding author(s): Liang Chen, NCOMMS-19-14129A

Last updated by author(s): Dec 10, 2019

Reporting Summary

Nature Research wishes to improve the reproducibility of the work that we publish. This form provides structure for consistency and transparency in reporting. For further information on Nature Research policies, seeAuthors & Referees and theEditorial Policy Checklist .

Statistics

For all statistical analyses, confirm that the following items are present in the figure legend, table legend, main text, or Methods section.

n/a	Confirmed	
☐	☒	The exact sample size (n) for each experimental group/condition, given as a discrete number and unit of measurement
☐	☒	A statement on whether measurements were taken from distinct samples or whether the same sample was measured repeatedly
☐	☒	The statistical test(s) used AND whether they are one- or two-sided<br>*Only common tests should be described solely by name; describe more complex techniques in the Methods section.*
☐	☒	A description of all covariates tested
☐	☒	A description of any assumptions or corrections, such as tests of normality and adjustment for multiple comparisons
☐	☒	A full description of the statistical parameters including central tendency (e.g. means) or other basic estimates (e.g. regression coefficient) AND variation (e.g. standard deviation) or associated estimates of uncertainty (e.g. confidence intervals)
☐	☒	For null hypothesis testing, the test statistic (e.g. F, t, r) with confidence intervals, effect sizes, degrees of freedom and P value noted<br>*Give P values as exact values whenever suitable.*
☒	☐	For Bayesian analysis, information on the choice of priors and Markov chain Monte Carlo settings
☒	☐	For hierarchical and complex designs, identification of the appropriate level for tests and full reporting of outcomes
☒	☐	Estimates of effect sizes (e.g. Cohen's d, Pearson's r), indicating how they were calculated

Our web collection on statistics for biologists contains articles on many of the points above.

Software and code

Policy information about availability of computer code

Data collection	TCGA and cBioPortal datebases
Data analysis	Image Lab software (version:3.0), Image J software (version:1.6), Flow Jo (version:7.6.1), Photoshop (version:CS5), SPSS software 19, Microsoft Excel 2017, Graphpad Prism 7.

For manuscripts utilizing custom algorithms or software that are central to the research but not yet described in published literature, software must be made available to editors/reviewers. We strongly encourage code deposition in a community repository (e.g. GitHub). See the Nature Research guidelines for submitting code & software for further information.

Data

Policy information about availability of data

All manuscripts must include a data availability statement. This statement should provide the following information, where applicable:

- Accession codes, unique identifiers, or web links for publicly available datasets
- A list of figures that have associated raw data
- A description of any restrictions on data availability

The data can been obtained from the TCGA datebase (https://portal.gdc.cancer.gov) and cBioPortal datebase (http://www.cbioportal.org).
Unprocessed gel blot of Figures are provided in Source date file.
All data supporting the findings in this study are available from the corresponding author upon reasonable request. We have provided a full data availability statement in the manuscript.

Field-specific reporting

Please select the one below that is the best fit for your research. If you are not sure, read the appropriate sections before making your selection.

- [x] Life sciences
- [ ] Behavioural & social sciences
- [ ] Ecological, evolutionary & environmental sciences

For a reference copy of the document with all sections, see nature.com/documents/nr-reporting-summary-flat.pdf

Life sciences study design

All studies must disclose on these points even when the disclosure is negative.

Sample size	Animal experimental studies were with a higher number of animals (n=8/group) than calculated by our standard power analysis. The statistical significance was assessed using two-tailed Student's t-test.
Data exclusions	No data were excluded from analysis.
Replication	Experiments in the article are reliably produced, replication were described in the figure legends.
Randomization	Animals with similar age and weight were randomly allocated to experimental groups.
Blinding	Investigators were blinded to group allocation during data collection and/or analysis.

Reporting for specific materials, systems and methods

We require information from authors about some types of materials, experimental systems and methods used in many studies. Here, indicate whether each material, system or method listed is relevant to your study. If you are not sure if a list item applies to your research, read the appropriate section before selecting a response.

Materials & experimental systems

n/a	Involved in the study	
[ ]	[x]	Antibodies
[ ]	[x]	Eukaryotic cell lines
[x]	[ ]	Palaeontology
[ ]	[x]	Animals and other organisms
[x]	[ ]	Human research participants
[x]	[ ]	Clinical data

Methods

n/a	Involved in the study	
[x]	[ ]	ChIP-seq
[ ]	[x]	Flow cytometry
[x]	[ ]	MRI-based neuroimaging

Antibodies

Antibodies used	Antibodies were obtained from Cell Signaling Technology: BiP (#3183, 1:1000), JNK (#9252, 1:1000), Phospho-SAPK/JNK (Thr183/Tyr185) (#9251, 1:1000), eIF2α (#9722, 1:1000), Phospho-eIF2α (Ser51) (#9721, 1:1000), cleaved caspase-3 (Asp175) (#9661, 1:1000), Keap1 (P586) (#4678, 1:1000); from Sigma: Flag M2 (F3165, 1:2000), tubulin (T6074, 1:2000); from MBL: GFP (M048-3, 1:1000), His (D291-3, 1: 3000); from Thermo Fisher Scientific: HA (SG77) (71-5500, 1:1000); from Novus Biologicals: TRIM25 (NBP2-20710, 1:1000); from Santa Cruz Biotechnology: Nrf2 (A-10) (sc-365949, 1:1000); from Proteintech: KEAP1 (10503-2-AP, 1:200) and Nrf2 (16396-1-AP, 1:200); from Abclonal: TRIM25 (A12938, 1:200); from Cell Signaling Technology: anti-rabbit IgG, HRP-linked Antibody (7074, 1:3000) and anti-mouse IgG, HRP-linked Antibody (7076, 1:3000);from Invitrogen: Goat anti-Mouse IgG, Alexa Fluor 488 (A-11001, 1:500) and Donkey anti-Mouse IgG, Alexa Fluor 568 (A-10037, 1:500) .
Validation	BiP antibody, human and mouse, WB, product citations:127 (https://www.cst-c.com.cn/products/primary-antibodies/bip-antibody/3183); JNK antibody, human and mouse, WB, product citations:1436 (https://www.cst-c.com.cn/products/primary-antibodies/sapk-jnk-antibody/9252); Phospho-SAPK/JNK (Thr183/Tyr185) antibody, human and mouse, WB and IP, product citations:1306 (https://www.cst-c.com.cn/products/primary-antibodies/phospho-sapk-jnk-thr183-tyr185-antibody/9251); eIF2α antibody, human and mouse, WB, product citations:393 (https://www.cst-c.com.cn/products/primary-antibodies/eif2a-antibody/9722); Phospho-eIF2α (Ser51) antibody, human and mouse, WB, product citations:454 (https://www.cst-c.com.cn/products/primary-antibodies/phospho-eif2a-ser51-antibody/9721); cleaved caspase-3 (Asp175) antibody, human and mouse, WB, IP, IHC, IF and F, product citations:3927 (https://www.cst-c.com.cn/products/primary-antibodies/cleaved-caspase-3-asp175-antibody/9661); Keap1 (P586) antibody, human and mouse, WB and IP, product citations:5 (https://www.cst-c.com.cn/products/primary-antibodies/keap1-p586-antibody/4678); Flag M2 antibody, all, WB, IP, IHC, IF and F, product citations:3325 (https://www.sigmaaldrich.com/catalog/product/sigma/f3165?lang=zh&region=CN); tubulin antibody, human and mouse, WB and IP, product citations:783 (https://www.sigmaaldrich.com/catalog/product/sigma/t6074?lang=zh&region=CN); GFP antibody, human and mouse, WB, IP, IHC and IF, product citations:36 (http://www.mbl-chinawide.cn/search-details2?id=1662&table=RuoAntibody); His antibody, human and mouse, WB, IP, F and IF, product citations:22 (http://www.mbl-chinawide.cn/search-details2?id=551&table=RuoAntibody); TRIM25 antibody, human, WB, IP, IHC and IF, validation using the TRIM25 knockout A549 cell lysate (https://www.novusbio.com/products/trim25-antibody_nbp2-20710#supportresearch); Nrf2

antibody, human, WB, IP and IF, product citations:9 (https://www.scbt.com/p/nrf2-antibody-a-10?requestFrom=search). KEAP1 antibody, human and mouse, WB, IP, IHC, IF, CoIP and ELISA, product citations:158 (http://www.ptgcn.com/products/KEAP1-Antibody-10503-2-AP.htm). Nrf2 antibody, human and mouse, WB, IP, IHC, IF, FC and ELISA, product citations:178 (www.ptgcn.com/products/NFE2L2,NRF2-Antibody-16396-1-AP.htm). TRIM25 antibody, human and mouse, WB, IP and IHC, product citations:1(https://abclonal.com.cn/catalog/A12938). Anti-rabbit IgG, HRP-linked antibody, human and mouse, WB, IP, IHC and ELISA, product citations:4146 (https://www.cst-c.com.cn/products/secondary-antibodies/anti-rabbit-igg-hrp-linked-antibody/7074); Anti-mouse IgG, HRP-linked antibody, human and mouse, WB, IP, IHC and ELISA, product citations:2216 https://www.cst-c.com.cn/products/secondary-antibodies/anti-mouse-igg-hrp-linked-antibody/7076);Goat anti-Mouse IgG, Alexa Fluor 488, Flow, ICC, IF and IHC, product citations:667 (https://www.thermofisher.com/cn/zh/antibody/product/Goat-anti-Mouse-IgG-H-L-Cross-Adsorbed-Secondary-Antibody-Polyclonal/A-11001). Donkey anti-Mouse IgG, Alexa Fluor 568, citations:32(https://www.thermofisher.com/cn/zh/antibody/product/Donkey-anti-Mouse-IgG-H-L-Highly-Cross-Adsorbed-Secondary-Antibody-Polyclonal/A10037).

Eukaryotic cell lines

Policy information about cell lines

Cell line source(s)	Human cell lines HCT116, Huh7 , MCF7, U2OS, MDA-MB-231 and embryo kidney cell line HEK293T were obtained from Cell Bank of Shanghai Institutes of Biological Sciences, Chinese Academy of Sciences.
Authentication	All cell lines are commercial and no authentication has been conducted after purchase.
Mycoplasma contamination	All cell lines were tested to be mycoplasma negative.
Commonly misidentified lines (See ICLAC register)	No cell lines used in this study were found in the database of commonly misidentified cell lines that is maintained by ICLAC .

Animals and other organisms

Policy information about studies involving animals; ARRIVE guidelines recommended for reporting animal research

Laboratory animals	Male athymic BALB/c nude mice (6 weeks old, Shanghai Institute of Materia Medica, Chinese Academy of Sciences) were raised in specific pathogen-free conditions. Mice were housed with a 12-hour light/dark schedule at 25±1°C and were fed an autoclaved chow diet and water ad libitum.
Wild animals	No wild animals were used in this study.
Field-collected samples	No field-collected samples were used in this study.
Ethics oversight	All animal experiments were undertaken in accordance with relevant guidelines and regulations and were approved by the Institutional Animal Care and Use Committee at SIAT.

Note that full information on the approval of the study protocol must also be provided in the manuscript.

Flow Cytometry

Plots

Confirm that:

- [x] The axis labels state the marker and fluorochrome used (e.g. CD4-FITC).
- [x] The axis scales are clearly visible. Include numbers along axes only for bottom left plot of group (a 'group' is an analysis of identical markers).
- [x] All plots are contour plots with outliers or pseudocolor plots.
- [x] A numerical value for number of cells or percentage (with statistics) is provided.

Methodology

Sample preparation	For cell apoptosis assay, around 1×106 cells were suspended in 1×Binding Buffer and incubated with annexin V-FITC and propidium iodide (PI) at room temperature (25 °C) for 15 min.
Instrument	FACS Calibur2Lasers
Software	FlowJo
Cell population abundance	No sorting was performed
Gating strategy	Gating strategy is provided in the manuscript.

- [x] Tick this box to confirm that a figure exemplifying the gating strategy is provided in the Supplementary Information.
